# Death Attitudes and Death Anxiety Among Medical Interns After the 2020 Outbreak of the Novel Coronavirus

**DOI:** 10.3389/fpsyg.2022.698546

**Published:** 2022-06-03

**Authors:** Yiqing He, Tao Li

**Affiliations:** ^1^School of Education, Guangzhou University, Guangzhou, China; ^2^Department of Radiology, Shenzhen Maternity and Child Healthcare Hospital, Shenzhen, China

**Keywords:** medical students, death attitude, death anxiety, COVID-19, death education

## Abstract

This study investigates the *status quo* and influencing factors of death attitudes and death anxiety among medical interns in China as measured by the *Death Attitude Scale* and *Death Anxiety Scale* following the outbreak of “Novel Coronavirus Pneumonia” in China in early 2020. Results of this study show that under the influence of COVID-19, in terms of death attitude, medical interns scored the highest in neutral acceptance and the lowest in escape acceptance. There were significant differences in death attitude and anxiety among the groups with different backgrounds, including their families’ approaches to discussions of death, the number of funeral experiences, and other factors. There were two additional factors affecting attitudes that were related to the epidemic situation: whether the individual had participated in work to treat COVID-19 and whether their close friends or relatives (“cherished persons”) had been diagnosed with COVID-19. The study reveals the ways that the epidemic had an impact on death attitude and death anxiety.

## Introduction

A “death attitude” is an individual’s feelings relating to the death of the self or others. It is an evaluative and relatively stable internal psychological tendency held by an individual in response to death (Dezutter et al., 2009; Du et al., 2020). Early studies on the concept while influenced by a public aversion to death and as such tended to focus on negative attitudes toward death such as fear, anxiety, avoidance, or denial. Holcomb et al. (1993), by contrast, determined that an individual’s death attitude was complex, combining various positive and negative emotions. Wong et al. (1994) further elaborated and delineated a model of death attitudes consisting of five components, namely, *Fear of Death*, *Death Avoidance*, *Neutral Acceptance*, *Approach Acceptance*, and *Escape Acceptance*. Wong’s model is widely used to measure the death attitude of many different people (Gao P. et al., 2018; Haratiyan et al., 2019; Barnett et al., 2020). When Brudek et al. (2020) revised Wong’s death attitude scale, they obtained research results that were consistent with the original version. Wang et al. (2020) explored the reliability and validity of the Death Attitude Profile-Revised (DAP-R) in measuring the death attitude of Chinese college students and found that this scale has good reliability and validity when applied to Chinese college students.

Death anxiety refers to an emotional state, including fear, worry, and restlessness that appears when one is reminded of the inevitability of death or is threatened by death (Jiao et al., 2020). A commonly used death anxiety measurement tool is the Templer-Death Anxiety Scale (T-DAS; Templer et al., 1971). Sharif Nia et al. (2020) systematically reviewed the psychometric characteristics of the cross-cultural T-DAS to ensure that it remains an effective and reliable scale when evaluating death anxiety in differing cultural backgrounds. Their results, which included studies in Iran, the United States, Italy, China, Egypt, Spain, and Australia, demonstrated that the T-DAS had remained a reliable tool. It is still commonly used to capture the conscious experience of death anxiety. Most recently, Huang et al. (2019) used the T-DAS to investigate the death anxiety of nurses in oncology departments, showing that the scale continues to be a valid and reliable instrument.

Previous studies have shown that many factors, including religious beliefs, gender, funeral, and family experiences, can influence death attitudes and death anxiety. When Gielen (2006) studied the relationship between Buddhist philosophy and death attitudes, they found that Buddhist philosophy positively affected a person’s ability to cope with death anxiety and helped generate a more accepting attitude toward death. Hu’s (2010) research explains that Buddhism views death as a renewal of life. When it is understood that it is only a matter of time before death comes, there is no need to fear and worry. Confucianism, the philosophical basis of Chinese traditional culture, also regards death as part of the natural process of life, while Zhuangzi taught that as death happens naturally, no one can avoid or resist it. Zhuangzi’s view of life and death can help people feel calm rather than anguish when faced with death.

Wang and Li (2017) investigated the relationship between death attitude and gender in a sample of 1,058 Chinese college students, finding that the level of death anxiety of women is higher than that of men. Yin et al. (2020), who studied death anxiety and death attitude among people of Tibetan and Han nationality, discovered that individuals who attended more funerals had more acceptance of death. Those who had more open discussions about death in the family also exhibited lower individual anxiety. Lázaro-Pérez et al. (2020), meanwhile found that Spanish law enforcement officers who worked during the start of the COVID-19 pandemic had a fear of death score of 82.1%, which was higher than the general situation. The reasons for this high score were the lack of Individual Protection Equipment and high levels of emotional exhaustion. The population at the center of the present study, medical students, differ from the general population as they have received life education and death education in college. Medical students tend to have a higher degree of neutral acceptance of death and a certain understanding of death. They are more likely to accept death than ordinary people and have lower than average fear of death (Liu and Zhang, 2015).

As COVID-19 continues to threaten China, people from all socioeconomic classes have been prompted to discuss the issue of death. Medical students often have to face death during the course of their studies and training. These experiences and their attitudes toward death in those moments affect their learning, life attitudes, and future careers (Shi et al., 2019). The changes medical students experience in their lifestyles and learning environments when they enter clinical practice after completing theoretical studies, as well as the possible exposure to death events, may have a great impact on students’ mental state and thoughts (Asadpour et al., 2016). Nia et al. (2016) found that nurses and other health care workers are exposed to diseases, trauma, and violence and that their death anxiety scores may be influenced by age, self-integrity, physical problems, mental illness, religious belief, race, occupational stressors, personal death experience, and media.

According to Jeff Greenberg’s Terror Management Theory, self-esteem is an evaluation and feeling of personal value that encapsulates people’s experience of their sense of life meaning and value. Terror Management Theory includes believing in the correctness of one’s cultural outlook on the world. Due to the influence of Chinese traditional culture, talking about death is often a taboo subject for Chinese natives (Zhang, 2020). This aversion to talking about death can be present even amongst medical students, some of whom will feel uneasy and try to avoid situations in which they might face or think about death (Zeng et al., 2019). But in a hospital environment, medical interns cannot avoid confronting death. When a death occurs, many interns will experience a state of fear, shock, and loss, and feel negative emotions, such as depression and sadness, due to a lack of knowledge and insufficient psychological preparation for such a situation. Some interns even choose to give up on their medical studies after a death event as they are unable to deal with the experience of death (dos Santos and Bueno, 2011). Ma et al. (2020) carried out a comparative study on death attitudes and death anxiety among emergency nurses before and after attending death education and training. The results show that the death education courses that focus on ways of dealing with sudden death can promote positive changes in emergency nurses’ attitudes toward sudden death and thereby reduce their death anxiety. Medical schools should strengthen the death education provided to medical students to influence their attitudes toward hospice care and death in a positive direction.

Medical students’ attitudes toward death are of particular importance as they adapt from the campus environment to the complex clinical work environment. This study investigated levels of death anxiety and death attitudes among medical interns who navigated this career transition after the outbreak of COVID-19.

## Research Hypotheses

This study intends to explore the death attitudes and death anxiety levels among medical interns after the outbreak of COVID-19 and to examine whether COVID-19 has had an impact on death attitudes and death anxiety among this population. The study will examine the following three hypotheses:

1. The theory of terror management has indicated that individuals’ background and culture affect their attitudes toward death. We, therefore, assume that the scores of women’s death anxiety will be higher than those of men’s, while those with religious beliefs will score lower than those without religious beliefs. In addition, we assume that individuals whose families were more open about death will have lower death anxiety scores and that medical interns who have attended more funerals will have higher scores with respect to neutral acceptance of death.
2. Regarding the relationship between medical interns’ death attitude and COVID-19 (Liu and Zhang, 2015; Lázaro-Pérez et al., 2020), we assume that people who have worked with patients with COVID-19 have higher scores of neutral acceptance than those who have not worked in those settings. We further assume that those who have been infected with COVID-19 have higher scores of neutral acceptance of death than those who have not been infected with COVID-19.
3. We used stepwise regression analysis to analyze whether medical interns’ gender, religious beliefs, past funeral experience, family atmosphere with regard to death, and experience with COVID-19 could partially predict their death attitude and death anxiety.

## Method

### Participants

This study is based on questionnaires that were distributed to clinical medical students who have left school for clinical practice by the counselor of a medical university in Guangdong in June and July 2022. These students are, mainly, in the fifth year of undergraduate study or the third year of a master’s degree. The students were able to choose to voluntarily fill out the questionnaire or could choose not to complete it. The completed questionnaires were collected in July 2022. The study includes 382 college students (mean age = 25.47, *SD* = 3.16) from a medical college in Guangdong province of which 219 (57.3%) were male students, and 163 (42.7%) were female students.

### Ethical Approval Statement

This study has been approved by the Academic Ethics Committee of the Corresponding author’s institution (ethical code: 2020051506). I certify that I have read and understood the Institution’s Policy for Ethical Practice, and I will comply with the ethical principles of these documents. I will submit, as appropriate, a Report on Research Progress or Amendment of an Approved Project if there are significant changes to my research or an adverse incident, in addition to any time when the report for annual progress is due.

### Measures

This study adopted a revised version of the attitude toward death profile compiled by Wong et al. (1994) and translated by Liao (2000) known as the Death Attitude Profile-Revised Scale (DAP-R). This scale consists of a total of 32 assessment items. Four key elements are: *Fear of Death*, where individuals facing death experience fear, fright, and other negative thoughts and emotions; *Death Avoidance*, where individuals avoid discussions or thoughts related to death; *Neutral Acceptance*, where individuals believe death to be an integral part of life to be neither feared nor welcomed; *Approach Acceptance*, where individuals see death as a channel to an afterlife in which people will live a happy life after death; and *Escape Acceptance*, where individuals believe that death can end the pain of living and accept death to escape the pain of life due to a deeper fear of continuing to live than of dying. A 5-point Likert scale was used to score each measure. Each item was set as follows: strongly disagree, disagree, uncertain, agree, and strongly agree, which were counted as 1–5 points, respectively, increasing successively. Scores were calculated separately for each dimension. This scale (DAP-R) has been widely used in the investigation of attitudes toward death in China (Zhu and Shi, 2011; Du et al., 2020; Wang et al., 2020). In this study, Cronbach’s alpha of the DAP-R’s five different death attitudes was: 0.79, 0.82, 0.75, 0.84, and 0.81.

The second measure used in this study is the Chinese Templer Death Anxiety Scale (CT-DAS) which originated from the Templer Death Anxiety Scale (T-DAS). The 3-week retest reliability of the original English version of the scale is 0.83. Yang et al. (2012), with the consent of the original author, created a Chinese version of the measure according to the cross-cultural adaptation guidelines recommended by the Evidence-based Committee of the American College of Orthopaedic Surgeons. The Cronbach’s alpha of this version is 0.71. This scale consists of 15 true/false statements designed to measure death attitudes, with reverse coding of the 2nd, 3rd, 5th, 6th, 7th, and 15th items. This scale has been widely used in the investigation of attitudes toward death in China (Chen and Cai, 2020; Guo and Zhang, 2020; He et al., 2020). In this study, the Cronbach’s alpha of CT-DAS was 0.68.

This study also took into account participants’ gender and religious beliefs, as well as their number of funeral experiences, the ways the participants’ families usually talked about death, whether the individual participated in the work against COVID-19, and whether important people around the participant had been infected with COVID-19. The true-false format was used to establish participants’ religious beliefs, their participation in work against COVID-19, and whether important people around the participant had been infected with COVID-19. If participants have a religious belief, they are asked to specify the religion they believe in. Participants’ funeral experiences were rated on a 5-point scale ranging from 1 = no funeral experiences to 5 = more than four funeral experiences. A final 4-point scale was used to assess the ways in which participants’ families spoke about death, ranging from 1 = speaking openly of death to 4 = never speaking of death.

### Procedure

Participants completed and submitted the questionnaire online, and participants were paid 5 RMB. Data analysis was conducted using SPSS 23.0. This study will use descriptive analysis to analyze medical interns’ attitudes toward death and anxiety toward death, *t*-test and *F*-test to different demographic variables, and stepwise regression analysis to understand the factors that influence attitudes and anxieties toward death. Repeated answers or incomplete questionnaires were eliminated. Of the total 405 questionnaires collected in this study, 382 were valid, resulting in an effective recovery rate of 94.3%.

## Results

### Status Quo of Death Attitudes and Death Anxiety of Medical Interns

For the most part, medical interns’ attitudes toward death scored highest in terms of neutral acceptance and lowest in terms of escape acceptance, as seen in Table 1.

**TABLE 1:** Results of the death attitudes and death anxiety survey (*n* = 382).

Project	Number of Items	M ± SD
Death anxiety	15	6.62 ± 2.79
Fear of death	7	2.77 ± 0.68
Death avoidance	5	2.70 ± 0.74
Neutral acceptance	5	4.04 ± 0.61
Approach acceptance	10	2.73 ± 0.60
Escape acceptance	5	2.69 ± 0.76

### Effects of Different Variables on Death Attitudes and Anxiety of Medical Interns

#### Gender

An independent sample *t*-test was used to compare the scores of medical interns of different genders in death attitudes and anxiety. The scores of medical interns of different genders were statistically significant in the dimension of approach acceptance and death anxiety (*p* < 0.05), as seen in Table 2.

**TABLE 2:** Results of death attitude and anxiety in different sex.

Project	Gender		T	P	Cohen’s d
	Male (*n* = 219)	Female (*n* = 163)			
Death anxiety	6.21 ± 2.65	7.17 ± 2.88	–3.360	0.001	–0.34
Fear of death	2.79 ± 0.67	2.74 ± 0.71	0.673	0.502	–
Death avoidance	2.68 ± 0.75	2.73 ± 0.72	–0.698	0.486	–
Neutral acceptance	4.09 ± 0.50	3.97 ± 0.74	1.908	0.057	–
Approach acceptance	2.79 ± 0.52	2.66 ± 0.68	2.020	0.044	0.21
Escape acceptance	2.72 ± 0.77	2.64 ± 0.74	1.017	0.310	–

#### Religious Belief

An independent sample *t*-test was conducted on medical interns with or without religious belief in each dimension of attitude toward death (Table 3), with results showing that different groups have a statistical significance in each dimension (*p* < 0.05). All participants with religious beliefs stated their belief to be Buddhism.

**TABLE 3:** Results of death attitude and anxiety in different religious beliefs.

Project	Religious belief		T	P	Cohen’s d
	Yes (*n* = 122)	No (*n* = 260)			
Death anxiety	6.07 ± 2.86	6.88 ± 2.72	–2.656	0.008	–0.29
Fear of death	2.95 ± 0.70	2.68 ± 0.66	3.542	<0.001	0.39
Death avoidance	2.90 ± 0.75	2.61 ± 0.71	3.582	<0.001	0.39
Neutral acceptance	4.16 ± 0.52	3.78 ± 0.71	5.837	<0.001	0.61
Approach acceptance	2.98 ± 0.63	2.62 ± 0.54	5.731	<0.001	0.61
Escape acceptance	2.83 ± 0.73	2.62 ± 0.76	2.529	0.012	0.28

#### Number of Funeral Experiences

A univariate analysis of variance and pair-wise comparisons were conducted on the scores of death attitude and anxiety of medical interns with different funeral experiences (Table 4). The results indicated that the different groups have a statistical significance in the dimension of neutral acceptance (*p* < 0.05).

**TABLE 4:** Results of a comparison of death attitude and anxiety of different funeral experiences.

Project	① (*n* = 92)	② (*n* = 77)	③ (*n* = 72)	④ (*n* = 46)	⑤ (*n* = 95)	F	P	Comparing
DAS	6.09 ± 2.56	6.40 ± 2.97	6.84 ± 2.82	7.06 ± 2.88	6.92 ± 2.74	1.63	0.166	–
DAP-R1	2.89 ± 0.63	2.86 ± 0.73	2.73 ± 0.69	2.69 ± 0.68	2.64 ± 1.68	2.10	0.080	–
DAP-R2	2.81 ± 0.72	2.70 ± 0.81	2.65 ± 0.72	2.63 ± 0.73	2.66 ± 0.72	0.698	0.594	–
DAP-R3	3.89 ± 0.65	4.01 ± 0.58	4.01 ± 0.74	4.30 ± 0.45	4.11 ± 0.53	3.80	0.005	① < ④
DAP-R4	2.80 ± 0.60	2.76 ± 0.55	2.78 ± 0.63	2.68 ± 0.54	2.64 ± 0.62	1.05	0.382	–
DAP-R5	2.72 ± 0.70	2.75 ± 0.79	2.67 ± 0.75	2.66 ± 0.75	2.62 ± 0.80	0.356	0.839	–

*① No, ② once, ③ twice, ④ three times, ⑤ four times or more; pairwise comparisons were significant at the 0.01 level. DAS (Death anxiety); DAP-R1 (Fear of death); DAP-R2 (Death avoidance); DAP-R3 (Neutral acceptance); DAP-R4 (Approach acceptance); DAP-R5 (Escape acceptance).*

#### The Way Families Talked About Death

A univariate analysis of variance was performed for each dimension of the attitudes toward death scale to consider differences in medical interns’ families’ discussions about death (Table 5). The results indicate statistically significant scores for each dimension of the attitudes toward death scale for each group.

**TABLE 5:** Statistical results of different the way families talked about death.

Project	① (*n* = 133)	② (*n* = 70)	③ (*n* = 158)	④ (*n* = 21)	F	p	Comparing
DAS	6.00 ± 2.93	6.25 ± 2.69	6.45 ± 2.92	7.24 ± 2.73	3.56	0.014	① < ④
DAP-R1	2.61 ± 0.67	2.80 ± 0.75	2.87 ± 0.65	2.92 ± 0.71	3.84	0.010	① < ②③④
DAP-R2	2.52 ± 0.68	2.78 ± 0.81	2.79 ± 0.74	2.94 ± 0.65	4.29	0.005	① < ②③④
DAP-R3	4.07 ± 0.65	4.14 ± 0.47	4.04 ± 0.56	3.48 ± 0.90	6.77	<0.001	① > ④
DAP-R4	2.70 ± 0.61	2.72 ± 0.61	2.75 ± 0.57	2.85 ± 0.70	0.501	0.681	–
DAP-R5	2.62 ± 0.78	2.64 ± 0.76	2.74 ± 0.73	2.84 ± 0.77	0.900	0.441	–

*① Naturally open talk; ② talk, but the atmosphere is not comfortable; ③ when necessary to talk about; ④ don’t talk about it. Pairwise comparisons were significant at the 0.05 level. DAP-R1 (Fear of death); DAP-R2 (Death avoidance); DAP-R3 (Neutral acceptance); DAP-R4 (Approach acceptance); DAP-R5 (Escape acceptance).*

Notably, the fear of death score was significantly lower among medical interns whose families mentioned death more naturally and more openly as compared to the scores of medical interns whose families mentioned death in an uncomfortable atmosphere.

#### Whether the Medical Student Has Participated in the Work Against COVID-19

An independent sample *t*-test for the dimensions of death anxiety and death attitudes was conducted for medical interns who had worked with COVID-19 patients (Table 6). The results indicated that the different groups have statistically significant differences in the dimensions of neutral acceptance and approach acceptance (*p* < 0.05).

**TABLE 6:** Results of death attitude and anxiety in participated in the work against COVID-19.

Project	Participated in the work against COVID-19		T	P	Cohen’s d
	Yes (*n* = 160)	No (*n* = 222)			
Death anxiety	6.61 ± 2.67	6.63 ± 2.88	–0.063	0.950	–
Fear of death	2.81 ± 0.67	2.74 ± 0.70	0.941	0.347	–
Death avoidance	2.74 ± 0.72	2.67 ± 0.75	0.911	0.363	–
Neutral acceptance	4.16 ± 0.16	3.95 ± 0.78	3.218	0.001	0.37
Approach acceptance	2.81 ± 0.56	2.68 ± 0.61	2.229	0.026	0.22
Escape acceptance	2.77 ± 0.76	2.62 ± 0.75	1.941	0.053	–

#### Whether Significant Persons (Family Member/Friend/Colleague/Neighbor) Have Ever Been Diagnosed With COVID-19

An independent sample *t*-test was performed on all dimensions of the attitudes toward death scale to compare the results of those medical interns whose family, friends, colleagues, or neighbors had been previously diagnosed as COVID-19 positive with those who did not know anyone diagnosed with COVID-19 (Table 7). The results indicated statistically significant differences in three dimensions of the measure: fear of death, death avoidance, and neutral acceptance. Scores for neutral acceptance were highest in medical students whose important persons had been diagnosed with COVID-19.

**TABLE 7:** Results of death attitude and anxiety in significant persons (family member/friend/colleague/neighbor) have ever been diagnosed with COVID-19.

Project	Have ever been diagnosed as COVID-19		T	P	Cohen’s d
	Yes (*n* = 76)	No (*n* = 306)			
Death anxiety	7.00 ± 2.87	6.52 ± 2.76	1.317	0.189	–
Fear of death	2.45 ± 0.66	2.85 ± 0.67	–4.559	<0.001	–0.60
Death avoidance	2.51 ± 0.78	2.75 ± 0.72	–2.486	0.013	–0.31
Neutral acceptance	4.67 ± 0.09	3.88 ± 0.59	11.559	<0.001	1.87
Approach acceptance	2.67 ± 0.71	2.75 ± 0.56	–1.008	0.314	–
Escape acceptance	2.73 ± 0.85	2.67 ± 0.73	0.632	0.528	–

### Regression Analysis of Factors Influencing Death Attitudes and Anxieties Among Medical Interns

Scores for *Death Anxiety, Fear of Death, Death Avoidance, Neutral Acceptance, Approach Acceptance*, and *Escape Acceptance* were used as dependent variables. Six factors additional factors—gender, religious beliefs, number of funeral experiences, the way families talked about death, whether the participant had worked with COVID-19 patients, and whether important persons in their life had been diagnosed with COVID-19—served as independent variables. A multiple stepwise regression method was used (Table 8).

**TABLE 8:** Summary of stepwise regression analysis of various influencing factors of participants.

	*R*	*R* ^2^	Δ*R*^2^	*F*	Δ*F*	*B*	β
**Death anxiety**							
Intercept						5.544	
Gender	0.170	0.029	0.029	11.288**	11.288**	1.026	0.182
religious belief	0.233	0.054	0.025	10.877***	10.194**	0.890	0.149
the way families talked about death	0.270	0.073	0.019	9.920***	7.626**	–0.393	–0.138
**Fear of death**							
Intercept						2.368	
Whether significant persons (family member/friend/colleague/neighbor) have ever been diagnosed with COVID-19	0.228	0.052	0.052	20.782***	20.782***	0.340	0.197
the way families talked about death	0.278	0.077	0.025	15.848***	10.400**	0.099	0.140
religious belief	0.307	0.094	0.017	13.097***	7.086**	–0.173	–0.117
number of funeral experiences	0.322	0.104	0.009	10.881***	3.930*	–0.045	–0.099
**Death avoidance**							
Intercept						2.895	
religious belief	0.181	0.033	0.033	12.828***	12.828***	–0.268	–0.168
the way families talked about death	0.239	0.057	0.024	11.478***	9.830**	0.119	0.157
**Neutral acceptance**							
Intercept						6.552	
Whether significant persons (family member/friend/colleague/neighbor) have ever been diagnosed with COVID-19	0.510	0.260	0.260	133.621***	133.621***	–1.053	–0.680
Whether the medical student has participated in the work against COVID-19	0.659	0.435	0.175	145.775***	117.103***	–0.554	–0.442
religious belief	0.678	0.460	0.025	107.146***	17.328***	0.191	0.144
Gender	0.686	0.471	0.011	83.870***	8.047**	–0.135	–0.108
**Approach acceptance**							
Intercept						3.689	
religious belief	0.282	0.080	0.080	32.849***	32.849***	–0.391	–0.304
Gender	0.316	0.100	0.020	21.036***	8.568**	–0.169	–0.139
Whether the medical student has participated in the work against COVID-19	0.336	0.113	0.013	16.051***	5.573*	–0.139	–0.114
**Escape acceptance**							
Intercept						3.918	
Religious belief	0.129	0.017	0.017	6.397*	6.397*	–0.251	–0.154
Whether the medical student has participated in the work against COVID-19	0.164	0.027	0.012	5.258**	4.068*	–0.240	–0.156
Whether significant persons (family member/friend/colleague/neighbor) have ever been diagnosed with COVID-19	0.198	0.039	0.010	5.150**	4.826*	–0.238	–0.125

*p < 0.05, **p < 0.01, and ***p < 0.001.

For *Death Anxiety*, gender, religious belief and family treatment of death entered the regression equation. The common explanatory quantity of the three factors (*R*^2^) was 7.3%, and the changes in *F* value were 11.29, 10.19, and 7.63, respectively, all reaching a significance level of < 0.01. The predictive power of death anxiety was 2.9, 2.5, and 1.9% respectively.

For *Fear of Death*, by contrast, the factors that were included in the regression equation were whether the participant knew people diagnosed with COVID-19, their family attitudes toward death, religious belief, and the number of funeral experiences. The common explanation amount of the four factors (*R*^2^) was 10.4%, and the changes in *F* values were 20.78, 10.40, 7.09, and 3.93, with a significance level of < 0.05. Predictive powers for *Fear of Death* were 5.2, 2.5, 1.7, and 0.9%.

For *Death Avoidance*, religious belief and family treatment of death were included in the regression equation. The common explanation amount of the two items (*R*^2^) was 5.7%, the changes of *F* values were 12.83 and 9.83, with significance levels of <0.01, and predictive powers of *Death Avoidance* being 3.3 and 2.4%.

The regression equation for *Neutral Acceptance* included four factors, namely, whether important persons had been diagnosed with COVID-19, whether subjects participated in the work against COVID-19, religious belief, and gender. This resulted in a common explanation (*R*^2^) of 47.1%, with predictive powers of 26.0, 17.5, 2.5, and 1.1%, and changes to the *F* value of 133.62, 117.10, 17.33, and 8.05, with a significance level of <0.01.

Religious belief, gender, and whether subjects had worked with COVID-19 patients, were included in the regression equation for *Approach Acceptance*. These three factors produced a common explanation amount (*R*^2^) of 11.3%, and changes to *F* values of 32.85, 8.57, and 5.57, with a significance level of <0.05. The predictors of individuals’ approaching acceptability to death were 8.0, 2.0, and 1.3%.

Finally, for *Escape Acceptance*, three factors, religious belief, whether subjects participated in the work against COVID-19, and whether important persons had been diagnosed with COVID-19, were included in the regression equation. The common explanation amount of the three factors (*R*^2^) was 3.9%, while the amount of change to *F* values were 6.40, 4.07, and 4.83, achieving a significance level of <0.05. The predictive power of an individual’s *Escape Acceptance* is 1.7, 1.2, and 1.0%, respectively.

## Discussion

Overall, Chinese medical interns show a moderate degree of death anxiety. This finding is consistent with the results of previous self-reported questionnaires (Deng et al., 2019). In terms of death attitudes, the results of this study suggest that the average score of the neutral acceptance dimension is slightly higher than the median value of 3. The result of this study is higher than the score found by Gao R. et al. (2018). The reason for this difference in score may be found in the experience of the pandemic. Due to COVID-19, medical interns are being exposed to more death events than was typical among medical interns 2 years prior. As such, medical interns who are working during the pandemic are able to build a more positive attitude toward death and are less inclined to try to escape death. The escape acceptance score was the lowest of all dimensions in the scale, indicating that most medical interns do not share the notion that death should be a means to alleviate pain. In the current COVID-19 pandemic, medical interns may be building greater mental fortitude than given the current higher occurrence of death.

The results of this study also indicate that participants’ gender, religious beliefs, prior funeral experiences, family attitudes toward death, experience working with COVID-19 patients, and possible knowledge of people diagnosed with COVID-19 caused significant differences in each dimension of the attitude toward death scale. In terms of gender differences, women have a higher level of death anxiety than men. The factors influencing this effect still need further investigation, but it is possible that women are more inclined to express their emotions than men, while men are more inclined to suppress their emotions. Moreover, women are more sensory-oriented, while men are more cognitive-oriented, with the result that men pay more attention to death, but express fewer anxious emotions around it (Jiang, 2016). When patients infected with COVID-19 die in hospital, female medical students’ death anxiety will be higher, perhaps because women’s psychological stress can be stronger than men’s (Zhu et al., 2020). In addition, the death anxiety of female medical students raised slightly when patients who had contracted COVID-19 died at the hospital. Medical students with religious beliefs scored higher in accepting death, and their anxiety concerning death was lower. The questionnaire recorded that the majority of participants believed in Buddhism, which teaches that no person can escape from birth to death and that since the material world is simply a process of existence, nothing can last forever. Buddhism further teaches that samsara is the expression of the flow of life and that after death our consciousness enters various new environments through the force of birth and produces different life forms. Although medical interns with religious beliefs have a high degree of acceptance of death neutrality, in the context of the COVID-19 pandemic, which featured more frequent death events and a shortage of medical protective equipment, the degree of fear of death and death avoidance of medical interns is still relatively high.

Stambrook and Parker (1987) suggested that when adults discuss death-related events, the atmosphere of discussion they create will affect their children’s attitudes toward death. This study showed that medical students whose families had a more natural and open atmosphere when discussing death were more likely to have a lesser fear of death and a more natural acceptance of death. This finding is consistent with the research results of Yang et al. (2018). Due to the outbreak of the COVID-19 epidemic, medical interns may face deaths every day. While their resilience may be tested by these events, they are likely to result in a greater acceptance of death.

Medical students who had attended more than four funerals scored significantly lower on the fear of death and death avoidance dimensions when compared to the other groups. This shows that the experience of attending a funeral can help to reduce negative attitudes such as fear of death and death avoidance. This result is consistent with Harrawood et al.’s (2009) survey. Medical interns who had participated in the work against COVID-19 and those who knew people diagnosed with COVID-19 showed a higher degree of neutral acceptance as compared to those who had not. The stepwise regression analysis indicated that previously working with COVID-19 patients and knowledge of people diagnosed with COVID-19 influenced each dimension of medical interns’ attitudes toward death. The results further suggested that the outbreak had predictive power.

To conclude, the death anxiety of medical interns remains at a moderate level. In the COVID-19 epidemic, however, the scores for fear of death and escape from death among this population are on the high side. Medical universities should strengthen death education for medical students and provide psychological protection courses during this sudden epidemic. These courses can explore the psychological and social impacts of death, provide professional theoretical knowledge, and establish a positive view of death in the process of discussion. Such training will help students make correct guidance and treatment decisions for patients and their families in their future clinical work. Given that medical students with Buddhist beliefs have a higher degree of neutral acceptance of death, students with Buddhist beliefs can be selected to lead and carry out various forms of death education such as internship, reading, films, and visiting patients (Yang et al., 2018).

### Limitations

A shortcoming of this study lies in the fact that the sampling of participants is limited to students studying in a certain medical college in Guangdong province who have already practiced in clinical practice. This sampling method affects the representativeness of participants. Furthermore, the use of an online questionnaire survey could not avoid the shortcomings of the questionnaire itself because it was impossible to observe each subject directly and record the reaction of the subject when answering questions. The explicit attitudes toward death reported by the participants could not reflect the real and complete individual attitudes toward death experienced internally. Medical interns’ implicit attitudes toward death should be explored by an experimental method in the future.

## Conclusion

During the COVID-19 epidemic, medical interns tend to accept death naturally. In general, they have a neutral acceptance of death. The factors influencing death attitude and death anxiety among medical interns included whether they had participated in the work against COVID-19 and whether they knew people diagnosed with COVID-19. In addition, the study shows that current attitudes toward death can be predicted according to six factors: gender, religious belief, prior participation in work against COVID-19, family atmosphere concerning the discussion of death, whether or not any cherished people had been diagnosed with COVID-19, and the number of instances of attending a funeral.

## Data Availability Statement

The raw data supporting the conclusions of this article will be made available by the authors, without undue reservation.

## Ethics Statement

The studies involving human participants were reviewed and approved by the City University of Macau. The patients/participants provided their written informed consent to participate in this study. Written informed consent was obtained from the individual(s) for the publication of any potentially identifiable images or data included in this article.

## Author Contributions

YQH was responsible for manuscript writing and data analysis. TL was responsible for data collection. Both authors contributed to the article and approved the submitted version.

## Conflict of Interest

The authors declare that the research was conducted in the absence of any commercial or financial relationships that could be construed as a potential conflict of interest.

## Publisher’s Note

All claims expressed in this article are solely those of the authors and do not necessarily represent those of their affiliated organizations, or those of the publisher, the editors and the reviewers. Any product that may be evaluated in this article, or claim that may be made by its manufacturer, is not guaranteed or endorsed by the publisher.
